# Electrochemical evaluation of porous CaFe_2_O_4_ anode material prepared via solution combustion synthesis at increasing fuel-to-oxidizer ratios and calcination temperatures

**DOI:** 10.1038/s41598-022-07036-3

**Published:** 2022-02-23

**Authors:** Jacob Strimaitis, Samuel A. Danquah, Clifford Denize, Sangram K. Pradhan, Messaoud Bahoura

**Affiliations:** 1 Center for Materials Research, Norfolk State University, 700 Park Ave, Norfolk, VA 23504 USA; 2 Engineering Department, Norfolk State University, 700 Park Ave, Norfolk, VA 23504 USA

**Keywords:** Batteries, Supercapacitors, Synthesis and processing

## Abstract

The drawbacks of common anodes in lithium-ion batteries (LIBs) and hybrid supercapacitors (HSCs), such as the high voltage plateau of Li_4_Ti_5_O_12_ (1.55 V vs. Li/Li^+^) and the moderate capacity of graphite (372 mAh-g^-1^), have established a need for better materials. Conversion materials, and in particular iron oxide and CaFe_2_O_4_ (CFO), have amassed recent attention as potential anode replacements. In this study, we evaluate the material and electrochemical effects of the solution combustion synthesis (SCS) of porous CFO across novel fuel-to-oxidizer ratios and calcination temperatures. We demonstrate that nearly doubling the amount of fuel used during synthesis increases capacities between 120 and 150% at high current densities (~ 1000 mA-g^-1^) and across 500 additional charging-discharging cycles, an effect brought on in part by enhanced compositional purity in these samples. However, in order to ensure long-term cyclic stability, it is necessary to also calcine porous CFO to 900 °C to enhance crystallite size, particle size and spacing, and compositional purity.

## Introduction

The longstanding consensus among scientists globally is that, as the energy demand continues to increase over the next few decades, so too will greenhouse gas emissions that significantly contribute to climate change and environmental decline^1–5^. These facts spur the need to shift away from fossil fuels as sources of energy and, instead, invest in developing and integrating a more diverse portfolio of cleaner and renewable sources of energy. Key to this modernization of our energy infrastructures, from large-scale power grids to small-scale portable electronics, is electrochemical energy storage (EES)^6–8^. In particular, Li-ion batteries (LIBs) have dominated the EES market for high energy applications, whereas supercapacitors (SCs) have covered high power applications^9–13^. Hybrid supercapacitors (HSCs), which incorporate materials of both LIBs and SCs together in one device, have also gained recent popularity, due to their relatively high energy and power densities, strong cyclabilities, and large voltage windows, among other advantages^14,15^. As fundamental building blocks for future energy systems, concerted efforts must be made to reduce the costs and increase the functionality of these EES devices, and most critical to this process is carefully selecting their material constituents.

LIBs and HSCs are made of a cathode and anode arranged on opposite sides of an electrolyte^15,16^. While their cathode materials often differ, LIBs and HSCs tend to require similar anode materials with particular anodic properties, such as large capacities, low voltage plateaus, and reversible chemical stabilities^17–19^. Of the three main types of anode materials, insertion-, conversion-, and alloy-type^20^, the most widely used for LIBs and HSCs are insertion-type materials, which rely on bulk penetrations of Li^+^ ions to induce Faradaic charge transfers at specific voltages^21,22^. The most common insertion-type anode materials are Li_4_Ti_5_O_12_ (LTO) and graphite^19,22,23^. However, these anodic frontrunners are not without their faults. For instance, despite strong Li^+^ intercalation/deintercalation reversibility, minuscule volume change during charging-discharging, and negligible solid electrolyte interphase (SEI) formation, LTO suffers from a relatively high voltage plateau at 1.55 V vs. Li^+^/Li, a relatively low theoretical capacity at 175 mAh-g^-1^, and low Li^+^ diffusion coefficients (< 10^–6^ cm^2^-s^-1^) and electronic conductivities (10^–13^ Ohm^-1^-cm^-1^) that affect rate performance^18,24^. Graphite has a lower voltage potential (0.1–0.2 V vs. Li/Li^+^) and a higher theoretical capacity (372 mAh-g^-1^) than LTO, making it a better candidate to increase the operational voltage window and energy densities, especially for HSCs^25–27^, but it also suffers from high current, rate capability, irreversible capacity, and cycle life issues^19,26,28^. Thus, there is a need to develop new battery-type anode materials that meet or exceed the capabilities of LTO, graphite, or other Li-ion insertion-type electrodes.

One promising type of material recently proposed to meet the performance needs for next-generation LIBs and HICs is that of conversion/displacement^29,30^, which rely on chemical transformations as a result of Li^+^ ion interaction^31^. These materials have lower working potentials (< 1 V vs. Li/Li^+^) and higher theoretical capacities (> 600 mAh-g^-1^) than most insertion-type anodes, high capacity reversibility, and, when carefully selected or modified, high power capabilities^29,30,32^. Owing to their high capacities, natural abundance, eco-friendliness, and cheap production cost, iron oxides (e.g., Fe_2_O_3_ and Fe_3_O_4_), in particular, have amassed attention as auspicious conversion/displacement anodes, though they are not free from issues of pulverization, detachment, and capacity loss due to volume changes^33,34^. Efforts to reach higher capacities at high rates and with better reversibility than graphite by mixing iron oxides with carbon additives have proven successful^35,36^, but even these solutions can be criticized for their complexity. An alternative option is to make composites with inactive alkaline earth metals^37^. Metal oxides enriched with Ca, for instance, form CaO “spectator” nanoparticles after the initial discharge process that buffer volume expansion and ultimately improve rate performance and cyclic stability^38,39^. In fact, the efficacy of this strategy was recently demonstrated by Han et al*.*^34^ when porous CaFe_2_O_4_ (pCFO; theoretical capacity 745 mAh-g^-1^) prepared via a simple sol–gel synthesis method showed considerable rate performance and cyclic stability compared to Fe_2_O_3_, an improvement that was ascribed to the CaO spectator nanoparticles found only in the pCFO sample.

Despite creating pCFO with excellent electrochemical performance, the sol–gel method of synthesis takes time and uses toxic materials^34,40^. To alleviate these issues, Shaji et al*.*^39^ synthesized pCFO via the quicker and eco-friendlier solution combustion synthesis (SCS) method. The SCS method is a well-known technique used to quickly and easily produce a variety of ceramic powders for a number of applications^41,42^. In particular, SCS can be employed to create nanoscale materials for electrochemical energy storage purposes^43^, including V_2_O_3_^44^, MoO_3_^45^, LiMn_2_O_4_^46^, and Fe_2_O_3_/C nanocomposites^47^. Shaji et al*.*^39^ found that their pCFO created via SCS reached a specific capacity of 437 mAh-g^-1^ at the end of a 500 cycle test at 500 mA-g^-1^, which is lower in capacity than Han et al*.*’s^34^ similarly-tested sol–gel pCFO, but still impressive when accounting for overall consistent rate performance and cyclic stability (see Table S1 for comparison of studies). However, it has not yet been shown how modifying the SCS method may improve pCFO electrochemical performance.

Herein, we report a more detailed look at the ecological SCS method for preparing pCFO active material. In particular, we investigate how changing the fuel-to-oxidizer ratio (*ϕ*) before synthesis and calcination temperature after synthesis alter electrochemical performance. It is demonstrated that increasing both *ϕ* and calcination temperature are easy and effective ways to enhance the electrochemical performance of pCFO. Specifically, pCFO prepared at a fuel-rich *ϕ* = 1.325 and calcined at 900 °C shows the best overall electrochemical performance out of all samples with an initial discharge capacity of 995 mAh-g^-1^, a nearly 100% capacity recovery after 20 cycles of high current charging-discharging, and an ending discharge capacity of 435 mAh-g^-1^ after an additional 500 cycle stability test at ~ 1000 mA-g^-1^.

## Results and discussion

### Preparation

Porous CaFe_2_O_4_ (pCFO) was synthesized using Solution Combustion Synthesis (SCS). The SCS reaction progresses according to the following equation, with the Ca- and Fe-species as the oxidizers and glycine as the fuel^41,48,49^:

1 $$\begin{gathered} {\text{Ca}}\left( {{\text{NO}}_{{3}} } \right)_{{2}} \cdot {\text{4H}}_{{2}} {\text{O }} + {\text{ 2Fe}}\left( {{\text{NO}}_{{3}} } \right)_{{3}} \cdot {\text{9H}}_{{2}} {\text{O }} + \, \left( {{4}0/{9}} \right)\phi {\text{C}}_{{2}} {\text{H}}_{{5}} {\text{NO}}_{{2}} + { 1}0\left( {\phi - {1}} \right){\text{O}}_{{2}} \to \hfill \\ {\text{CaFe}}_{{2}} {\text{O}}_{{4}} + \, \left[ {\left( {{1}00/{9}} \right)\phi + { 22}} \right]{\text{H}}_{{2}} {\text{O }} + \, \left[ {\left( {{2}0/{9}} \right)\phi + { 4}} \right]{\text{N}}_{{2}} + \, \left( {{8}0/{9}} \right)\phi {\text{CO}}_{{2}} \hfill \\ \end{gathered}$$

**Figure 1:**
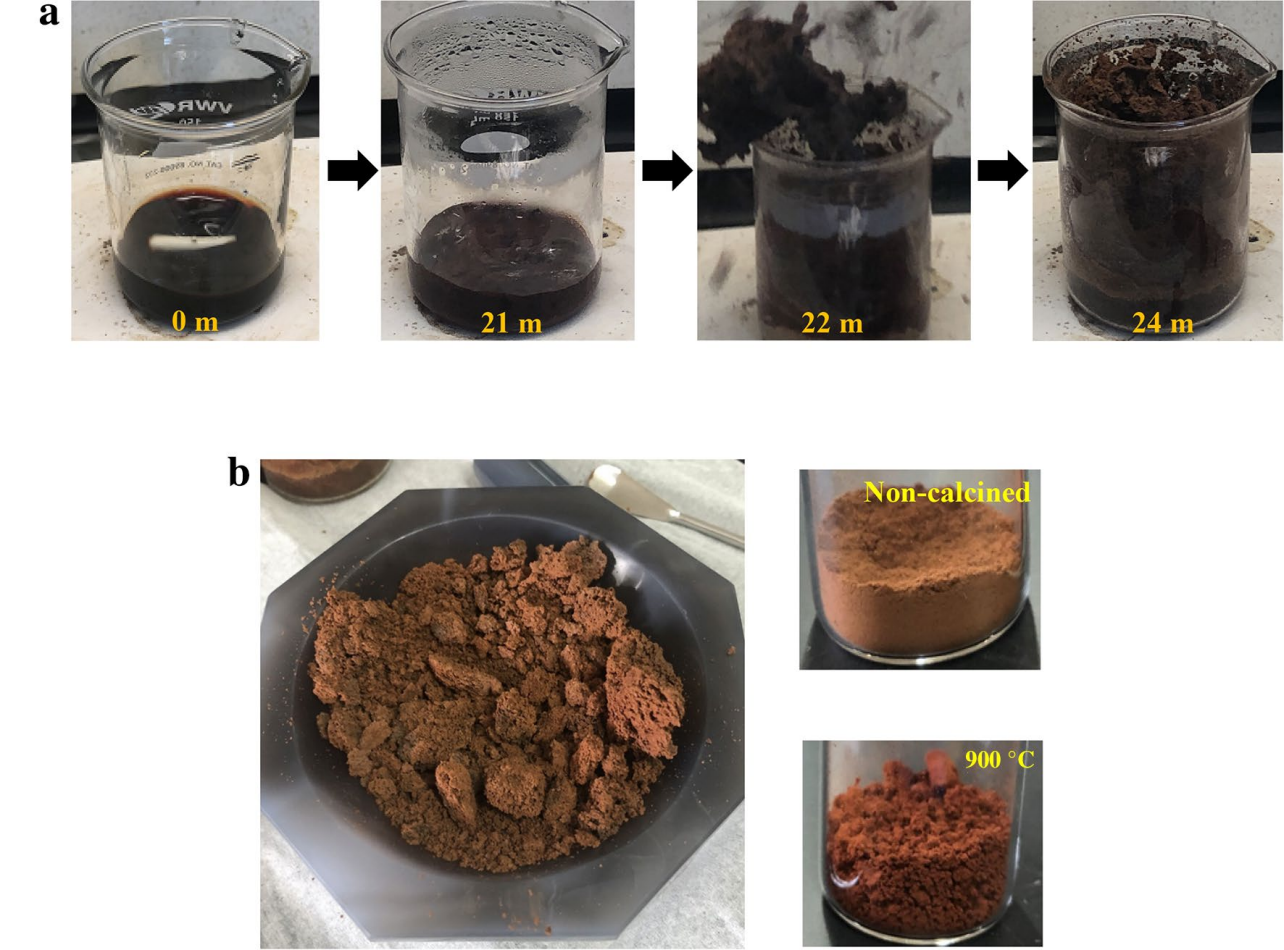
Preparation of porous CaFe_2_O_4_ (*ϕ* = 0.675) via Solution Combustion Synthesis: (**a**) timeline of synthesis by setting the hot plate surface temperature to 300 °C, (**b**) final product before grinding (left), after grinding (top-right), and after full calcination at 900 °C (bottom-right).

The symbol, *ϕ*, is the fuel-to-oxidizer ratio, which is a representation of the valences of the reducing species (glycine) with respect to the valences of the oxidizing species (metal nitrates). It is also used as a multiplication factor of the fuel in SCS equations. In this study, different batches of pCFO were synthesized at *ϕ*’s of 0.675 (fuel-lean condition), 1 (stoichiometrically balanced condition), and 1.325 (fuel-rich condition), which also correspond to the fuel-to-oxidizer mole ratios, *n*, of 1, 1.48, and 1.96, respectively. The chronology of a typically less than 30-min SCS reaction is depicted in Fig. 1a. The resulting foam is lightweight and easily grinds into a fine powder (Fig. 1b). When calcined, the powder changes both color and texture, turning into a crumbly brownish-red with increasing temperature.

### Material characterization

**Figure 2:**
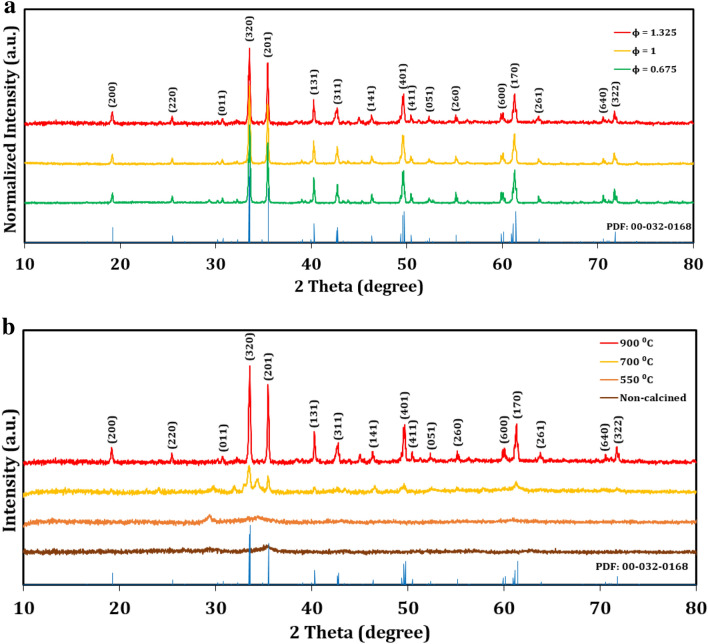
XRD patterns of CaFe_2_O_4_: (**a**) synthesized at increasing *ϕ* and calcined at 900 °C, (**b**) synthesized at *ϕ* = 1.325 and calcined at different temperatures. Spectra have been vertically scaled and offset for clarity.

The purity and crystallinity of the samples were evaluated using x-ray diffraction (XRD; Fig. 2). At full calcination (900 °C), the diffraction peaks match, both in position and relative intensity, with indexed orthorhombic CaFe_2_O_4_ (PDF card No. 00-032-0168), indicating strong phase purity and consistency between batched syntheses of pCFO across fuel-to-oxidizer ratios. Estimations of average crystallite size, based on the Scherrer equation^46^, are 107.9 nm, 96.5 nm, and 102.6 nm for samples prepared at *ϕ* = 0.675, 1, and 1.325, respectively, suggesting that increases in *ϕ* do not necessarily induce increases in crystallite size. Calcination temperature has a much more marked effect on average crystallite size, however, with sizes of 6.6 nm, 19.7 nm, 27.8 nm, and 102.6 nm corresponding to the no calcination temperature, 550 °C, 700 °C, and 900 °C samples, respectively. Increases in calcination temperature also correspond with increases in signal-to-noise ratio (Fig. 2b), further confirming the reports of others that crystallite size and crystallinity increase with calcination temperature^34,42,46,50,51^. In addition, we observe peaks at two theta (2θ) values of 34.4 in the 700 °C sample and 29.36 in the 550 °C sample that do not match with any significant peaks of orthorhombic CaFe_2_O_4_, indicating the existence of other as yet identified phases besides CaFe_2_O_4_ in these samples.

**Figure 3:**
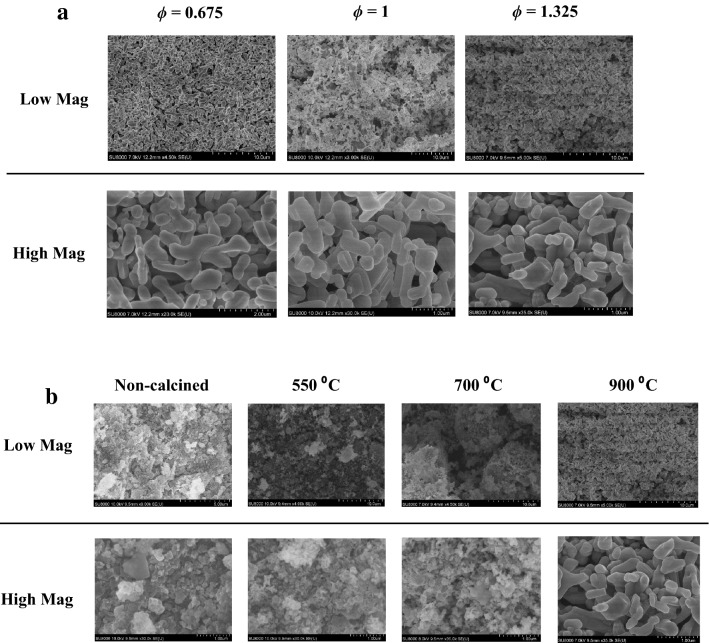
FESEM images of pCFO at low magnification (× 3–9 k) and high magnification (× 20–35 k): (**a**) images of pCFO synthesized at increasing *ϕ* and calcined at 900 °C, (**b**) images of pCFO synthesized at *ϕ* = 1.325 and calcined at different temperatures.

The morphology of pCFO was examined with field emission scanning electron microscopy (FESEM; Fig. 3). Much like the crystallinity comparison (Fig. 2a), the FESEM comparison of pCFO synthesized at increasing *ϕ* does not reveal any obvious morphological differences (Fig. 3a). Across the board, particles are randomly distributed, smooth, and shaped like oblong beans or Tic Tacs that range in length from 200 nm to 2 μm. Others have identified the porous nature of CaFe_2_O_4_ particles based on the recognition of macroporous (> 50 nm) voids in SEM images^34^, an observation that holds true for the pCFO particles in this study as well. Interestingly, there does not appear to be any significant interparticle connectivity or agglomeration, which is unlike the “chain-framework” of pCFO synthesized using a similar procedure^39^. More noticeable morphological differences emerge in the FESEM comparison of pCFO synthesized with a high proportion of glycine fuel (*ϕ* = 1.325) and increasing calcination temperatures (Fig. 3b). For all samples examined before full calcination at 900 °C, pCFO appears as fuzzy pieces of shale or coral-encased rock between 1 and 15 μm in diameter. Higher magnification inspections reveal smaller grains (50–200 nm) of material that dot the surface of the larger pieces, creating dense, nano-sized alcoves on the surface. It is only after full calcination at 900 °C that the morphology of pCFO dramatically increases in size or “popcorns” to the large, deeply macroporous, and oblong shapes of the increasing *ϕ* samples. The tendency for the particle size of SCS powders to generally increase with post-synthesis heat treatment is common and attributed to the congregation of crystallites^46,51^. Consistent, too, with this explanation is the observation of increasing crystallite size with calcination temperature (Fig. 2b). The lack of more obvious pCFO particle growth between 550 and 700 °C and the punctuated growth of smooth pCFO particles at some threshold temperature between 700 and 900 °C has, to our knowledge, not been reported and so may be unique for SCS-pCFO systems. However, even this unusual observation corresponds with the similarly stinted crystallite growth up to 700 °C and more dramatic crystallite growth between 700 and 900 °C observed in our crystallinity analysis (Fig. 2b), further bolstering the notion that crystallite size influences particle size.

**Figure 4:**
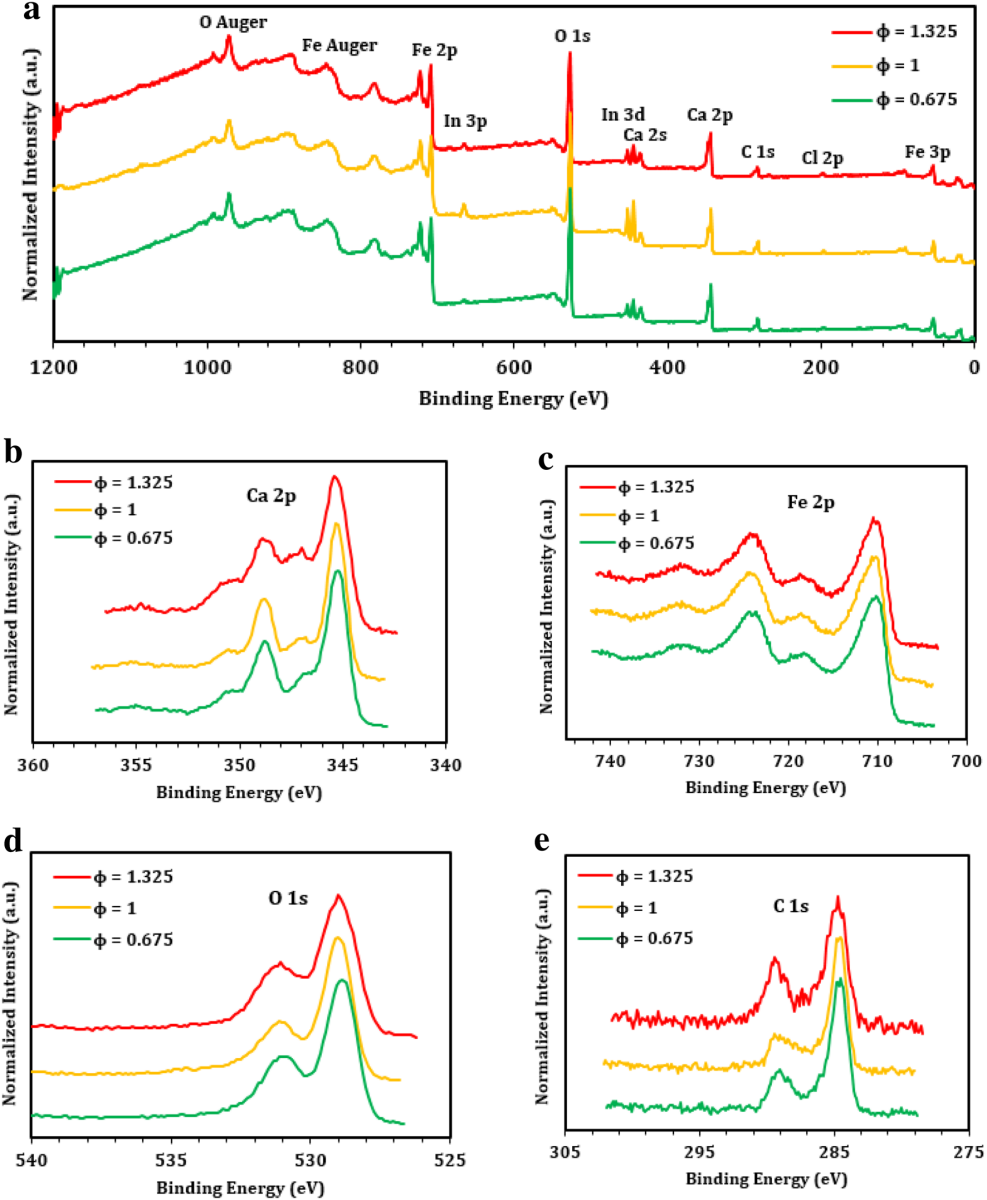
XPS spectra of pCFO synthesized at increasing *ϕ* and calcined at 900 °C: (**a**) survey scan, (**b**) Ca 2p scan, (**c**) Fe 2p scan, (**d**) O 1 s scan, and (**e**) C 1 s scan. Spectra have been normalized to global maxima and vertically offset for clarity.

**Figure 5:**
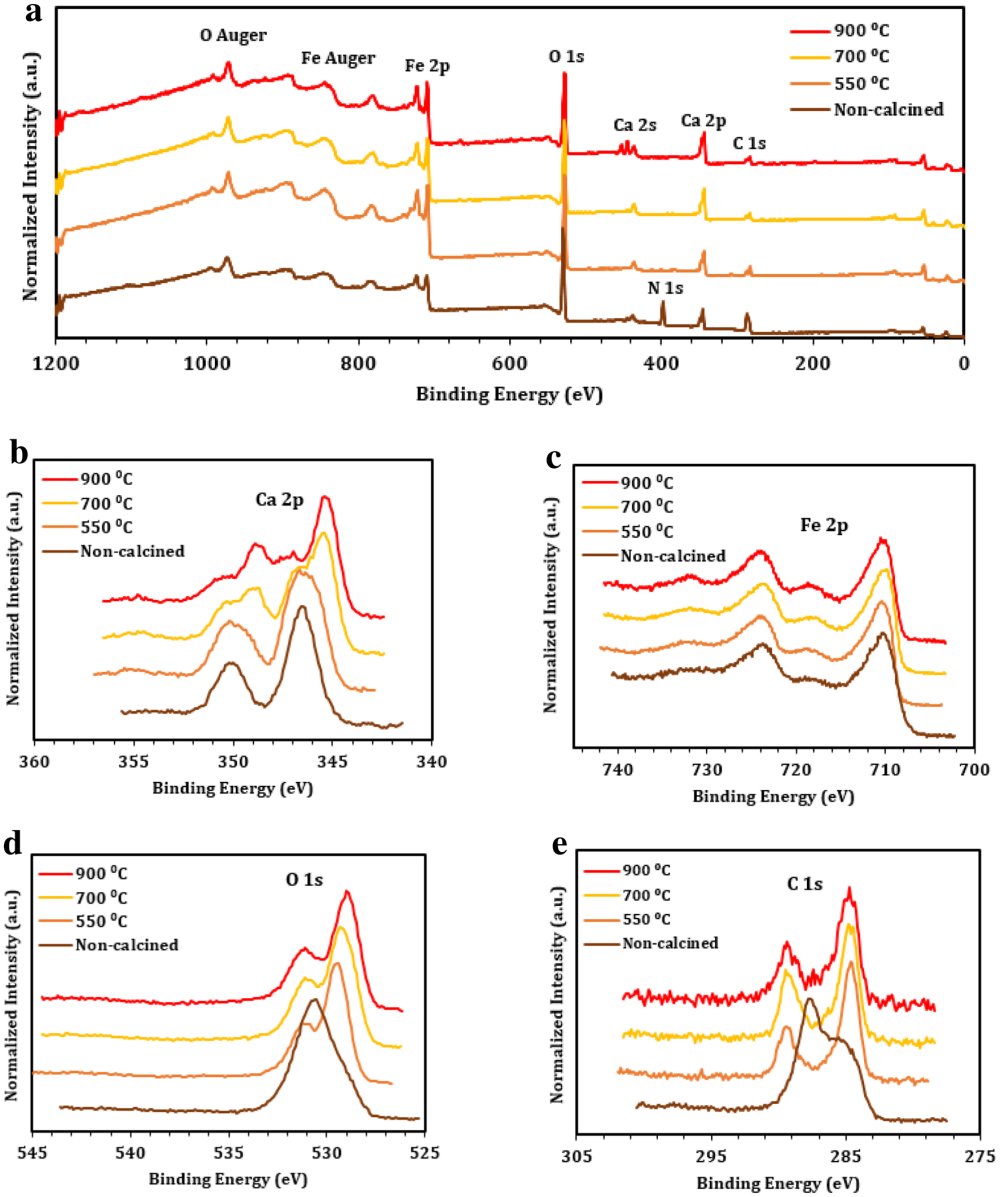
XPS spectra of pCFO synthesized at *ϕ* = 1.325 and calcined at different temperatures: (**a**) survey scan, (**b**) Ca 2p scan, (**c**) Fe 2p scan, (**d**) O 1 s scan, (**e**) C 1 s scan. Spectra have been normalized to global maxima and vertically offset for clarity.

X-ray photoelectron spectroscopy (XPS) was used to monitor the surface elemental composition of pCFO (Figs. 4, 5, and Table 1). Survey scans of pCFO synthesized at increasing *ϕ* and constant calcination temperature (900 °C) reveal the presence of the expected Ca, Fe, O, and C elements (Fig. 4a)^39^. The only unexpected element in all these scans, besides traces of substrate In, is Cl (Cl 2p doublet at ~ 200 eV and Cl 2 s at ~ 260 eV), which is likely the result of high-temperature environmental contamination. The Ca 2p spectra have two primary peaks at binding energies of 345.3 eV and 348.7 eV, corresponding to Ca 2p_3/2_ and Ca 2p_1/2_, respectively, of pCFO (Fig. 4b). The Ca 2p spectra also show the existence of lower intensity peaks at binding energies of 347.0 eV and 350.4 eV, corresponding to Ca 2p_3/2_ and Ca 2p_1/2_, respectively, of a secondary Ca-based oxide or hydroxide species, such as CaO, Ca(OH)_2_, or some non-stoichiometric CaFe_x_C_y_O_z_ species^52,53^. The average relative abundance of primary to secondary species is near 70/30 across *ϕ* (Figure S1). The Fe 2p spectra have two primary peaks at binding energies of 710.0 eV and 723.8 eV, corresponding to Fe 2p_3/2_ and Fe 2p_1/2_, respectively, and broad satellite peaks at binding energies of 718.1 eV and 732.0 eV that are characteristic of Fe in the 3^+^ oxidation state (Fig. 4c). The O 1 s spectra have one primary peak at a binding energy of 529.0 eV, most likely indicative of lattice oxygen, and a secondary peak at a binding energy of 531.0 eV, which most likely corresponds to surface hydroxyl groups (Fig. 4d)^52,53^. The C 1 s spectra have one primary peak corrected to a binding energy of 284.6 eV due to C–C and C=C species, and a secondary peak at a binding energy of 289.0 eV due to C=O species (Fig. 4e)^52,53^. These functional groups may come from residual carbon in the sample or adventitious carbon on the surface of the sample. As the fuel-to-oxidizer ratio increases during synthesis, the elemental compositions of pCFO samples approach the ideal Ca:Fe:O ratio of 1:2:4 (Table 1).

**Table 1:** Compositional ratios, reported as XPS-derived atomic percentages normalized to the Ca concentration, of primary and trace elements in pCFO across *ϕ* (calcination temperature = 900 °C) and across calcination temperature (*ϕ* = 1.325).

Compositional ratios based off atomic percent								
Element	Ideal	*ϕ*			Calcination temperature			
		0.675	1	1.325	None	550 °C	700 °C	900 °C
Ca	1	1	1	1	1	1	1	1
Fe	2	2.6	2.4	2.2	2.6	4.0	2.6	2.2
O	4	5.6	5.6	5.0	9.0	7.2	5.7	5.0
C	–	1.9	1.8	1.5	7.8	2.4	1.7	1.5
Cl	–	0.08	0.1	0.1	–	–	–	0.1
N	–	–	–	–	3.5	–	–	–

XPS survey scans of pCFO synthesized at the same fuel-to-oxidizer ratio (*ϕ* = 1.325) but increasing calcination temperature similarly reveal the presence of the expected Ca, Fe, O, and C elements in all samples (Fig. 5a)^39^. There are only two unexpected occurrences: the first is the Cl peaks in the fully calcined (900 °C) sample (Fig. 4a). The second is a sharp N 1 s peak at a binding energy of 400.0 eV in the as-synthesized sample only, indicating the presence of N in an organic matrix that appears to entirely “burn off” by the first calcination temperature of 550 °C^53^. The Ca 2p spectra show the existence of secondary Ca 2p doublet peaks at binding energies of 346.5 eV and 350.0 eV that transition to primary doublet peaks at binding energies of 345.3 eV and 348.7 eV as calcination temperature increases (Figs. 5b, S1), indicating the possible transition from a Ca-based oxide or non-stoichiometric oxide species to the intended CaFe_2_O_4_ species with increased temperature^52,53^. As with the increasing *ϕ* samples, the identity of this secondary Ca-based species is unclear. However, it is likely not residual Ca(NO_3_)_2_, since there are no N peaks in the calcined samples (Fig. 5a), nor are there any higher binding energy peaks indicative of Ca(NO_3_)_2_ in either the Ca 2p (Fig. 5b) or O 1 s spectra (Fig. 5d)^52,53^. The O 1 s spectra for calcined samples have one primary peak at binding energies between 529.0 and 529.5 eV and a secondary peak at a binding energy of 531.0 eV (Fig. 5d), indicating a similar oxygen composition in these samples as in the increasing *ϕ* samples. However, the as-synthesized sample has a very broad, asymmetric peak with a major component at a binding energy of 530.5 eV, consistent with hydroxides or other oxide species, and a smaller component at a binding energy closer to 529 eV that is similar to the oxide species found in the calcined samples^53^. The C 1 s spectra for calcined samples mimic those of the increasing *ϕ* samples with two adventitious carbon peaks at binding energies of 284.6 eV and 289.1 eV (Fig. 5e). Also, like with the O 1 s spectra comparison, the C 1 s spectrum of the as-synthesized sample shows a deviation from its calcined counterparts with a primary peak at a binding energy of 284.6 eV and a taller secondary peak at a binding energy of 287.5 eV, indicating a greater proportion of a new carbon species (albeit one still with C=O functionality) relative to the C–C and/or C=C adventitious carbon^53^. While the identity of this new carbon species in the non-calcined sample is unknown, it likely burns off upon calcination. As with the increasing *ϕ* samples, increasing calcination temperature generally changes the elemental compositions of pCFO towards the ideal Ca:Fe:O ratio of 1:2:4 (Table 1), particularly with marked declines in percent composition of O and C. Some residual O and C are certainly due to adventitious carbon. Taken together, as-synthesized pCFO begins as a disorganized array of Ca-based iron oxide or non-stoichiometric oxide species with significant amounts of adventitious C-N and carboxyl functional groups. After heating to 550 °C, all N-based groups have burned off, along with ~ 70% of other carbon contaminates and 20% of oxygen. With further calcination to 700 °C and even to 900 °C, more carbon, oxygen, and secondary Ca-based groups are removed until what remains, as was also evidenced in the increasing *ϕ* samples (Fig. 4), is a large presence of the primary CaFe_2_O_4_ species with only residual traces of adventitious carbon, a secondary Ca-based oxide, and some combination of other surface-level hydroxyl and/or carboxyl contaminates.

### Electrochemical performance

**Figure 6:**
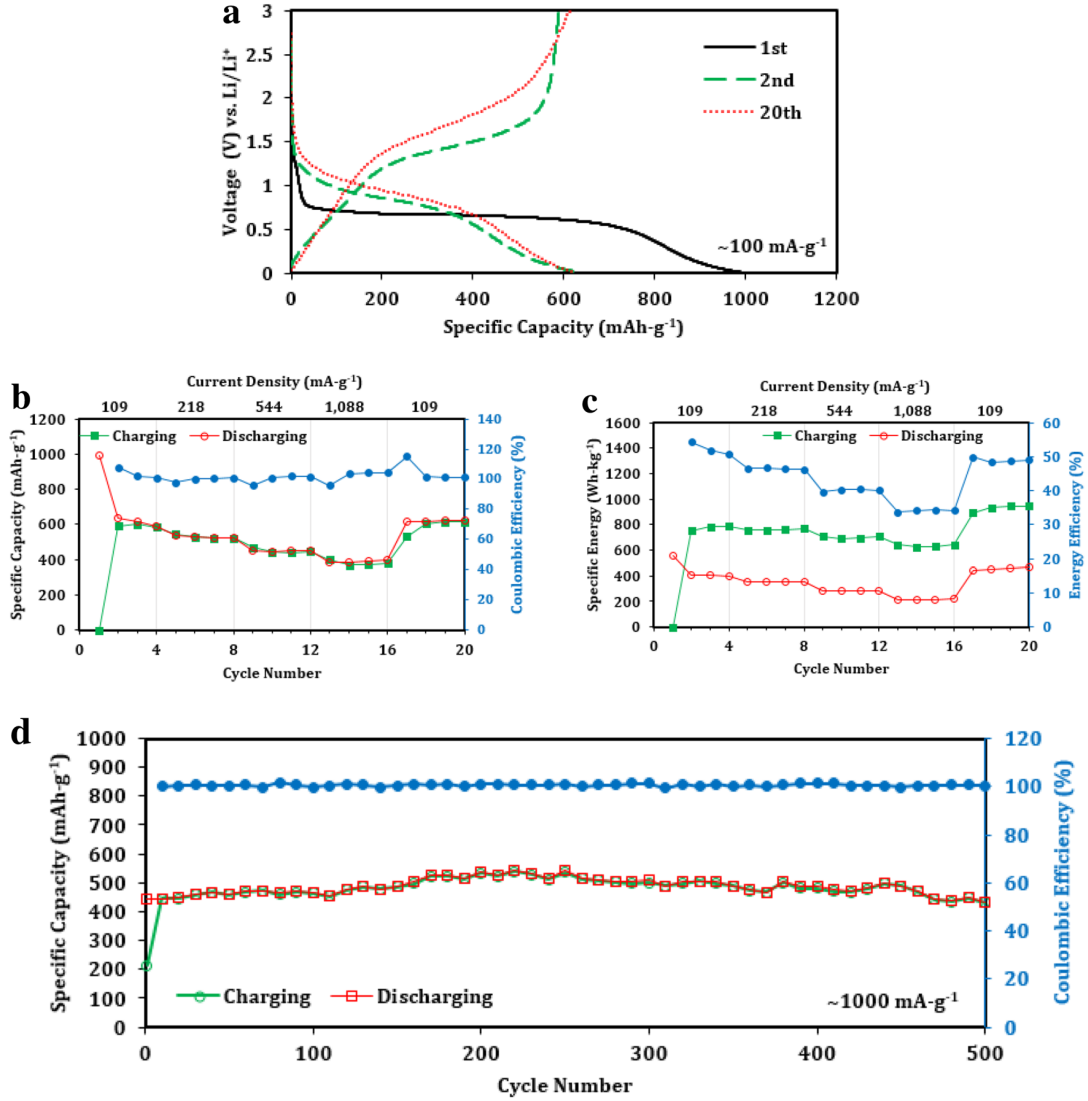
Electrochemical performance of best-performing porous CaFe_2_O_4_ anode (*ϕ* = 1.325, 900 °C calcination): (**a**) galvanostatic charge–discharge curves of 1st, 2nd, and 20th cycles at a current density of 109 mA-g^-1^; (**b**) rate performance in specific capacity; (**c**) rate performance in specific energy; (**d**) cycling stability at 1088 mA-g^-1^ up to additional 500 cycles after initial 20 cycle rate performance. Expectedly large initial efficiencies are omitted for clarity (Figure S2).

The galvanostatic charging-discharging showcase of the best-performing device, which was made with pCFO synthesized at a high fuel-to-oxidizer ratio (*ϕ* = 1.325) and fully calcined at 900 °C, demonstrates the powerful capabilities of this material as an electrode (Fig. 6). The first discharge plateaus at ~ 0.7 V vs. Li/Li^+^ and delivers a specific capacity of 995 mAh-g^-1^ at a current density of 109 mA-g^-1^ (Fig. 6a), which is comparable to other pCFO synthesized via SCS^39^. Mechanistically, this discharge corresponds to the lithiation of CaFe_2_O_4_ lattice structures and the irreversible creation of inactive CaO nanograins, according to Eq. (2)^32,54,55^:

2 $${\text{CaFe}}_{{2}} {\text{O}}_{{4}} + {\text{6e}}^{ - } + {\text{6Li}}^{ + } \to {\text{2Fe}}^{{\text{o}}} + {\text{3Li}}_{{2}} {\text{O}} + {\text{CaO}}$$

3 $${\text{2Fe}}^{{\text{o}}} + {\text{3Li}}_{{2}} {\text{O}} \leftarrow \to {\text{Fe}}_{{2}} {\text{O}}_{{3}} + {\text{6Li}}^{ + } + {\text{6e}}^{ - }$$

The subsequent charge at the same current density corresponds to the oxidation of metal iron particles and reduction/delithiation of Li_2_O between 1.3 and 1.7 V vs. Li/Li^+^, according to the forward reaction in Eq. (3). The second discharge drops in capacity from the initial discharge by 64% to 637 mAh-g^-1^, which is a widely-reported phenomenon in conversion materials known as irreversible capacity loss, or ICL, that is likely due to irreversible electrolyte decomposition, incomplete back conversions, and/or SEI formation^30,39^. This discharge also corresponds with the reversible conversion of Fe_2_O_3_ back to metal Fe and the reformation of Li_2_O, according to the reverse reaction in Eq. (3). By the 20th cycle in the rate performance test, the capacity has held mostly consistent at 621 mAh-g^-1^. Figure 6a also demonstrates known fallibilities inherent with conversion material’s charge–discharge performance^30^, such as the large kinetic hysteresis between charging and discharging (also represented as low energy efficiencies in Fig. 6c) and the relative sloping nature of the discharging “plateaus” after the first discharge.

The resiliency and stability of pCFO were evaluated with a rate performance test in which the device was cycled at the current densities of 109, 218, 544, and 1088 mA-g^-1^ (Fig. 6b). The discharge specific capacities corresponding to the last cycle of each of these current densities are 615, 526, 453, and 395 mAh-g^-1^, respectively. While the capacities expectedly decrease with the tenfold rise in current density, the lowest capacities of cycles 13–16 are still, on average, a formidable 63% of the average highest capacities of cycles 2–4. Discharge capacity recovery is also strong, maintaining an average of 100% of the discharge capacity between cycles 2–4 and 17–20.

Cyclic stability for pCFO is great (Fig. 6d), starting at 445 mAh-g^-1^, peaking to 542 mAh-g^-1^ at cycle 220, and falling back down to 435 mAh-g^-1^ at the end of the additional 500th cycle. Considering the device delivered capacities in the range of 382–395 mAh-g^-1^ at the same current density during the preceding rate performance test, the fact that capacities exceed this range over the subsequent 500 cycles is unexpected but indicative of the overall resiliency and stamina of this electrode.

**Figure 7:**
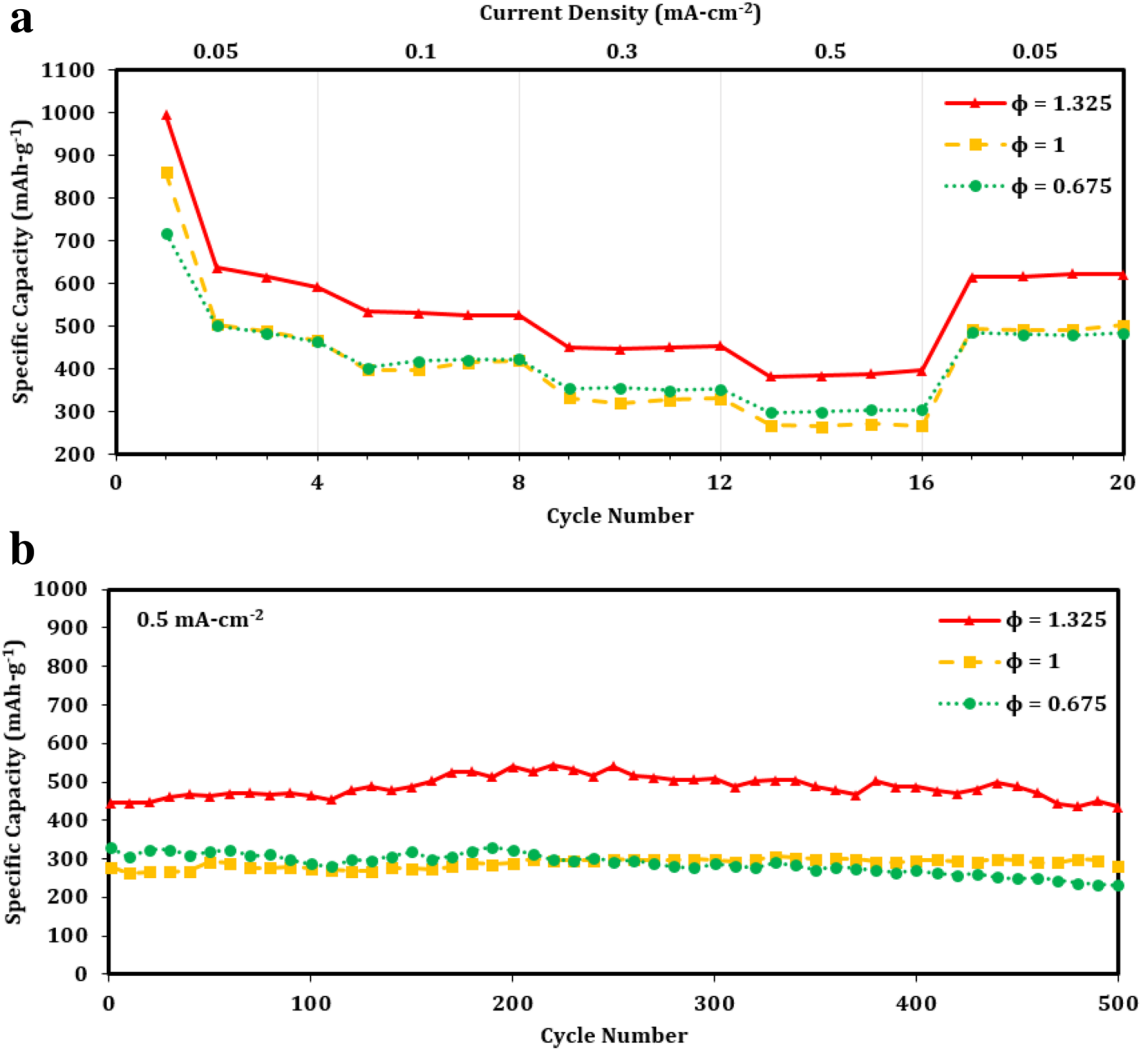

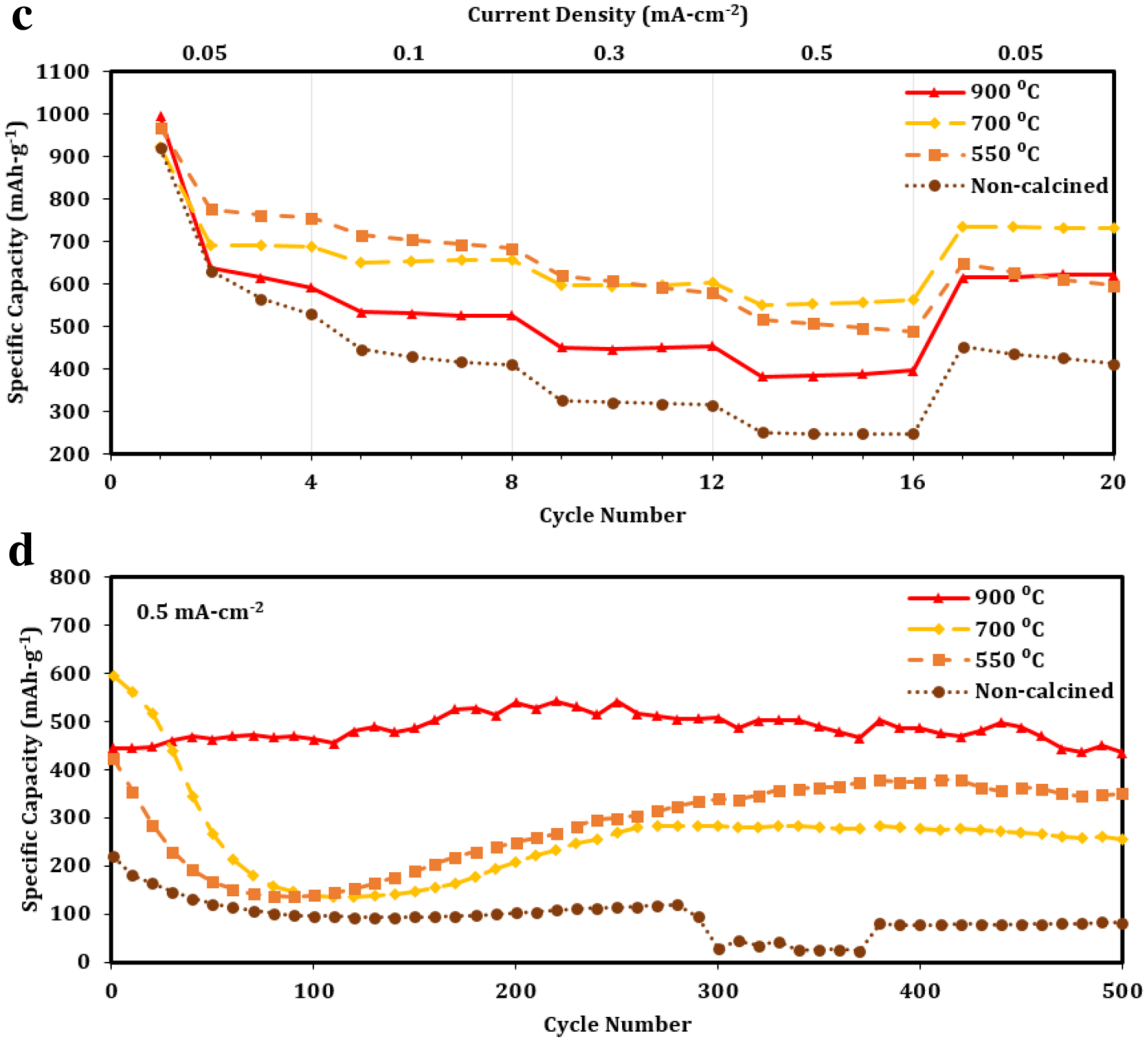
Electrochemical discharge performance of porous CaFe_2_O_4_ samples: (**a**) rate performance and (**b**) subsequent long-term cycling performance of pCFO synthesized at increasing *ϕ* and calcined at 900 °C; (**c**) rate performance and (d) subsequent long-term cycling performance of pCFO synthesized at *ϕ* = 1.325 and calcined at different temperatures.

The rate performance and long-term cyclic stability of all pCFO electrodes are compared in Fig. 7. Excluding current transitions and beginnings of tests, Coulombic efficiencies are consistently near 100% for all samples, demonstrating excellent reversibility (Figure S2). Changing the *ϕ* has the most noticeable effect on overall specific discharge capacity (Figs. 7a, b). For initial discharges, the capacities increase linearly by a factor of ~ 1.15 as *ϕ* increases (Fig. 7a). However, with subsequent cycling at higher currents, pCFO prepared in fuel-lean (*ϕ* = 0.675) and stoichiometrically balanced (*ϕ* = 1) conditions nearly overlap in capacity, whereas pCFO prepared in a fuel-rich (*ϕ* = 1.325) condition jumps up in capacity between 1.2 and 1.5 times its counterparts. This trend holds true for the subsequent cyclic stability tests, too, where capacities delivered by the fuel-rich pCFO are, on average, 1.2 times higher than the overlapped performance of pCFO prepared in lesser fuel conditions (Fig. 7b). A previous report with similar experimental conditions found that iron oxide prepared in a fuel-rich environment has greater longterm cycling capacity than the same material prepared in fuel-lean or stoichiometrically balanced conditions, an effect that was ascribed to the greater porosity, surface area, and conductive carbon generated in situ in the fuel-rich sample^47^. In the present study, however, we note no noticeable differences in interparticle spacing or morphology among samples prepared at increasing *ϕ* (Fig. 3a), and if anything, we see a decrease in carbon content across increasing *ϕ* samples (Table 1). There are also no clear phase or crystallinity differences (Fig. 2a). As such, the reason for the enhanced performance in our fuel-rich sample must lie elsewhere. One possibility, based on Table 1, is that the higher *ϕ* samples benefit from greater compositional purity and, therefore, an enhanced availability of lithiated CFO to generate charges according to Eq. (2). This explanation accounts for the linear increase in initial discharge capacity that mirrors, for example, the linear decrease in the composition of Fe with increasing *ϕ*. However, it does not fully address why the fuel-lean and stoichiometrically balanced samples do not show appreciable differences in capacity for the remainder of the rate performance and cyclic stability tests where Eq. (3) processes dominate. It might be the case that the subsequent benefits of compositional purity require some compositional threshold to be crossed, though this exact point is unknown at this time.

To evaluate the effectiveness of calcination temperature on pCFO prepared in fuel-rich (*ϕ* = 1.325) conditions, pCFO samples were heated to various temperatures after synthesis and then subjected to rate performance and subsequent cyclic stability tests. Based on the rate performance test alone (Fig. 7c), it is unclear which sample has the best performance. All samples start with relatively similar initial discharge specific capacities between 900 and 1000 mAh-g^-1^, but with additional cycling, a few differences emerge. The non-calcined sample shows the sharpest decline in capacity, followed by the lowest capacities of all temperatures across the five currents. The device with the next lowest capacities across the currents is the one with pCFO fully calcined to 900 °C, which are, on average, 1.1–1.6 times higher than the non-calcined pCFO. The best-performing devices are, surprisingly, the remaining pCFO samples partially calcined to 550 °C and 700 °C, with capacities closer to 1.5–2.2 times higher than the non-calcined pCFO device. While the 550 °C sample has the highest capacities in cycles 2–8, and so might initially appear to be the strongest between the remaining two, it shows a noticeable sloping decrease in capacity across cycles 13–16 and cycles 17–20, indicating a type of degradation at those currents that is not present in the 700 °C sample. The 700 °C sample also shows a stronger recovery and bounces back to an average of 733 mAh-g^-1^ across cycles 17–20, compared to an average of 690 mAh-g^-1^ across earlier cycles 2–4 at the same current.

With the accompanying 500 cycles cyclic stability test, though, it becomes more apparent that the fully calcined (900 °C) device has superior overall performance (Fig. 7d). The capacities for the partially calcined samples (550 °C and 700 °C) dramatically drop to 25–35% of their initial capacities around the 100th cycle and only rise to 83% and 43% of their initial capacities, respectively, by the end of the 500 cycles, whereas the capacity for the fully calcined sample (900 °C) floats between 97 and 122% of its initial capacity across all 500 cycles. The non-calcined sample shows the least stability, with a drop in capacity over the first 100 cycles that appears to stabilize over the next 180 cycles but drops dramatically between cycles 300–370 and only partially recovers for the remaining cycles. The dramatic drop and subsequent increase in capacity over the first 200 cycles for the partially calcined samples is a common feature in conversion materials, the explanation for which has been attributed to increases in metallic particle sizes/agglomeration during cycling that decreases the kinetic availability of Li^+^ during cycling^30^. By contrast, the purported reason that pCFO samples are relatively stable compared to most metal oxide conversion materials is that the CaO nanograins, which are released during the initial discharge, buffer volume expansion and prevent metallic agglomeration^34,39^. While it is unconfirmed why the partially calcined samples show the “conversion material cycling valley” by the 100^th^ additional cycle, morphological data indicate that their native pCFO nanograins may be too densely packed to allow penetration of CaO nanograins in the bulk where the prevention of metal iron particle agglomeration would be most effective (Fig. 3b). When pCFO is fully heated to 900 °C, its particles “popcorn” to a significantly larger size with both greater CaFe_2_O_4_ crystallite size and crystallinity (Fig. 2b) and interparticle spacing (Fig. 3b). These characteristics may be better suited for CaO nanograin creation and penetration and thus CaO-based stability.

## Conclusions

In summary, porous CaFe_2_O_4_ can be easily synthesized using solution combustion synthesis, and if certain parameters are adjusted during its preparation, such as the fuel-to-oxidizer ratio and calcination temperature, marked effects occur. Specifically, nearly doubling the amount of fuel for the synthesis raises specific discharge capacities by 120–150% across 500 additional cycles at high currents, an effect that may be explained by an improvement in compositional purity. While discharge capacities are noticeably higher across initial cycles at high currents for samples that were only partially calcined after synthesis, the best-performing sample of our study is fully calcined to 900 °C, as its greater crystallite and particle size, interparticle spacing, and compositional purity allow for an enhanced long term cyclic stability compared to its partially calcined counterparts. With an improved fuel-to-oxidizer ratio of 1.325 and calcination temperature of 900 °C, porous CaFe_2_O_4_ can reach an initial discharge capacity of 995 mAh-g^-1^ at ~ 100 mA-g^-1^, subsequent discharge capacities near 615 mAh-g^-1^ at ~ 100 mA-g^-1^ (with 100% capacity recovery after multiple high rate cycles), and excellent cyclic stability from 435 to 542 mAh-g^-1^ at ~ 1000 mA-g^-1^ across 500 additional charge–discharge cycles. Thus, porous CaFe_2_O_4_ prepared at a high fuel-to-oxidizer ratio and fully calcined shows great promise as a resilient, stable, and ultra-high-capacity anode material for lithium-ion battery and hybrid supercapacitor applications.

## Experimental

### Chemicals list

Calcium nitrate tetrahydrate (Ca(NO_3_)_2_ · 4H_2_O, ≥ 99.0%, Sigma-Aldrich), iron nitrate nonahydrate (Fe(NO_3_)_3_ ·  9H_2_O, ACS reagent, ≥ 99.0%, Sigma-Aldrich), glycine (C_2_H_5_NO_2_, USP & FCC grade, J. T. Baker), carboxymethyl cellulose sodium salt (CMC, n ~ 1050, Tokyo Chemical Industry), carbon black (acetylene black, 99.9 + %, Alfa Aesar), deionized water (DI H_2_O). All chemicals were used as-is unless otherwise stated.

### Active material synthesis

The active material, porous CaFe_2_O_4_ (pCFO), was prepared in a similar way to previous reports^39,49^. First, precalculated amounts of the precursor Ca-species, the Fe-species, and glycine were endothermically dissolved in a beaker with ~ 12 mL of DI H_2_O, placed on a stir/hot plate, and stirred at room temperature for 5 min with a magnetic stir bar spinning at 200 rpm. Next, the plate was heated to 250–300 °C with continuous stirring, starting the Solution Combustion Synthesis (SCS). After 10–15 min, the solution began to boil while maintaining mostly the same dark-red color, opaqueness, and liquid consistency as it had at room temperature. After another 10–15 min, the solution became a dark-brown, roiling batter, and within 3–5 more minutes, the rapid (< 3 s) self-combustion reaction occurred. The product was a dark, crumbly, low-density foam that easily ground into a terra-cotta powder. This powder was then calcined in air at different holding temperatures of 550, 700, or 900 °C (furnace ramping rate of 10 °C per minute) for 3 h, becoming lighter shades of brown to brownish-red with increasing temperature targets.

### Electrode preparation

Electrodes were prepared via a manual slurry mixing and deposition process. Active material (pCFO), carbon black (acetylene black), and carboxymethyl cellulose sodium salt (CMC; dissolved in DI H_2_O overnight at 12.5 mg-mL^-1^) were combined in a 7:2:1 mass ratio. First, the active material and carbon black powders were dry mixed for 5 min. Then, CMC was added dropwise, and the whole slurry was thoroughly ground with a mortar and pestle until the liquid had evaporated (~ 30 min). The dried material was then resuspended with additional DI H_2_O, mixed for 1–2 min, and deposited onto stainless steel spacers (15.5 mm diameter, MTI Corp.) while wet via a concentric circle painting technique. Active material mass loadings were between 0.4 and 0.7 mg-cm^-2^. After drying in air, electrodes were further dried in vacuum at 80 °C for 5 h.

### Coin cell preparation

Coin cells (type 2032) were assembled as half cells in an argon-filled glovebox (< 0.5 ppm H_2_O, < 0.5 ppm O_2_). Components were typically arranged as follows: cathode-side can, wave spring, pCFO electrode as cathode, separator (Celgard), electrolyte (50–80 μL of 1 M LiPF_6_ in EC/DMC 1:1; Sigma-Aldrich), Li chip as anode, stainless steel spacer (15.5 mm diameter × 0.5 mm thickness), and anode-side cap. Devices were manually crimped for a hermetical seal and then left alone to stabilize for at least 2 h before testing. Open circuit voltage of devices before testing was usually 2.5—2.7 V vs. Li/Li^+^.

### Material characterization

The phase and crystal information of powder samples were measured via x-ray diffraction (XRD) using a SmartLab X-ray Diffractometer, Rigaku, ran in the Bragg Brentano geometry with a Cu x-ray source. Morphology was observed via field emission scanning electron microscopy (FESEM; Hitachi SU8010). Composition and electronic states of sample surfaces were measured using x-ray photoelectron spectroscopy (XPS; Kratos Axis Ultra DLD, monochromatic Al Kα source). Samples were pressed into indium metal, and a charge neutralizer was used to minimize charging effects. Survey and high-resolution scans were acquired at pass energies of 80 eV and 20 eV, respectively. All XPS data were corrected to the C 1 s peak at 284.6 eV.

### Electrochemical characterization

Galvanostatic electrochemical performance was evaluated using an 8 Channel Battery Analyzer (MTI Corp.). Coin cells were placed in holders and linked up to the analyzer via alligator clips or inserted directly onto a testing board. All tests were between 0.01 and 3 V vs. Li/Li^+^ (charge up then charge down = 1 cycle) and proceeded in two sequential phases: rate performance and 500 cycle evaluation. For rate performance, cells were charged-discharged at 0.099, 0.198, 0.495, and 0.99 mA. There was a one-minute constant voltage (3 V) step between each charge and discharge. For subsequent 500 cycle evaluation, cells were charged-discharged 500 times at 0.99 mA with no constant voltage step. All densities were reported per unit mass of active material (pCFO). A comparison of active material masses and current densities, reported as mA-g^-1^, for each sample can be found in Table S2.

## Supplementary Information

Supplementary Information.
