# Supplementary material for: Electrochemical evaluation of porous CaFe2O4 anode material prepared via solution combustion synthesis at increasing fuel-to-oxidizer ratios and calcination temperatures
Source: Sci Rep. 2022 Feb 23;12:3082. doi: 10.1038/s41598-022-07036-3 (PMC8866530; doi:10.1038/s41598-022-07036-3)
Supplement: Supplementary file 1 — Supplementary Information. [file 41598_2022_7036_MOESM1_ESM.docx]

**Supporting Information**

**Electrochemical Evaluation of Porous CaFe_2_O_4_ Anode Material Prepared via Solution Combustion Synthesis at Increasing Fuel-to-oxidizer Ratios and Calcination Temperatures**

Jacob Strimaitis^1^, Samuel Danquah^1^, Clifford Denize^1^, Sangram K. Pradhan^1^, Messaoud Bahoura^1,2^

Norfolk State University, 700 Park Ave., Norfolk, VA 23504

^1^Center for Materials Research

^2^Engineering Department

^*^Corresponding authors:

Jacob Strimaitis [j.strimaitis@spartans.nsu.edu](mailto:j.strimaitis@spartans.nsu.edu)

Messaoud Bahoura [mbahoura@nsu.edu](mailto:mbahoura@nsu.edu)

**Table S1.** Comparison of electrochemical performance of pCFO.

|  | **Ref [1]** | **Ref [2]** | **ϕ (Calcined 900 ⁰C)** | | | **Calcination Temperature (ϕ = 1.325)** | | | |
| --- | --- | --- | --- | --- | --- | --- | --- | --- | --- |
| **Category** |  |  | **0.675** | **1** | **1.325** | **None** | **550 ⁰C** | **700 ⁰C** | **900 ⁰C** |
| **Highest Initial Discharge (mAh/g @ mA/g)** | ~900 @ 50 | 1050 @ 100 | 718 @ 74 | 861 @ 94 | 995 @ 109 | 919 @ 94 | 967 @ 129 | 922 @ 129 | 995 @ 109 |
| **Highest Second Discharge (mAh/g @ mA/g)** | ~560 @ 50 | 671 @ 100 | 501 @ 74 | 504 @ 94 | 637 @ 109 | 630 @ 94 | 775 @ 129 | 692 @ 129 | 637 @ 109 |
| **Lowest Discharge (mAh/g @ mA/g)** | 321 @ 1000 | 311 @ 5000 | 298 @ 744 | 265 @ 943 | 383 @ 1088 | 247 @ 943 | 488 @ 1286 | 551 @ 1286 | 383 @ 1088 |
| **End of Rate Performance Capacity Recovery (mAh/g @ mA/g)** | ~600 @ 50 | 733 @ 100 | 484 @ 74 | 502 @ 94 | 621 @ 109 | 412 @ 94 | 597 @ 129 | 731 @ 129 | 621 @ 109 |
| **Longterm Cycling Shape** | Flat | Flat | Flat | Flat | Small Hill | Dip-plateau | Dip-plateau | Dip-plateau | Small Hill |
| **~500 Cycles (mAh/g @ mA/g)** | 437 @ 500 | ~450 @ 1000 | ~288 @ 744 | ~288 @ 943 | ~487 @ 1088 | 29-220 @ 943 | 137-432 @ 1286 | 136-594 @ 1286 | ~487 @ 1088 |

[1] Shaji, N., Santhoshkumar, P., Nanthagopal, M., Senthil, C. & Lee, C. W. Electrochemical performance of porous CaFe2O4 as a promising anode material for lithium-ion batteries. *Appl. Surf. Sci.* **491**, 757–764 (2019).

[2] Han, C. *et al.* Porous CaFe2O4 as a promising lithium ion battery anode: A trade-off between high capacity and long-term stability. *Nanoscale* **10**, 12963–12969 (2018).

**Figure S1.** High-resolution Ca 2p spectra showing peak fitting for pCFO powders, and relative concentrations of the two Ca species.


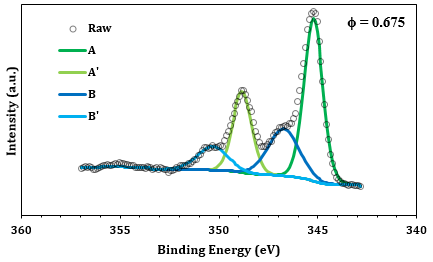


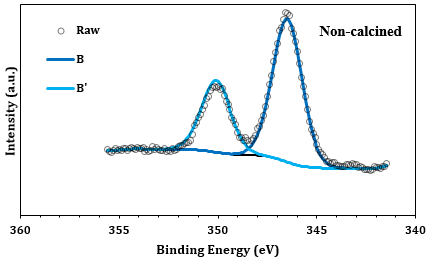


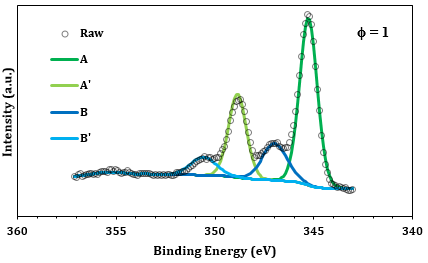


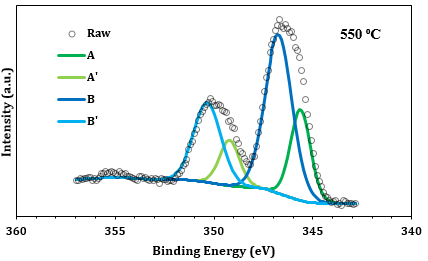


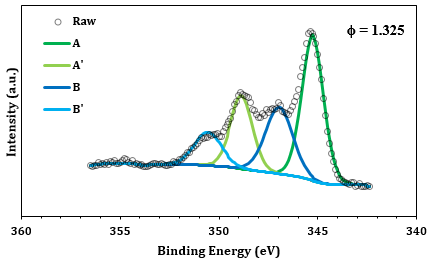

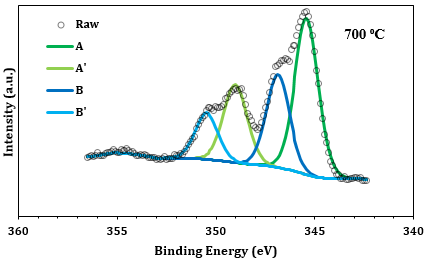


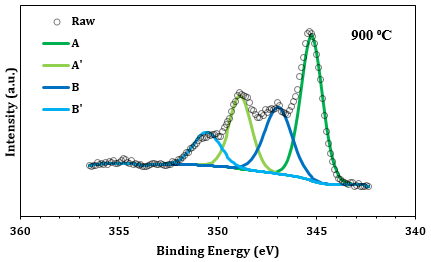


| **Relative concentration of two Ca species** | | | | | | | |
| --- | --- | --- | --- | --- | --- | --- | --- |
|  | **ϕ (Calcined 900 ⁰C)** | | | **Calcination Temperature (ϕ = 1.325)** | | | |
| **Peaks** | **0.675** | **1** | **1.325** | **None** | **550 ⁰C** | **700 ⁰C** | **900 ⁰C** |
| **A+A’** | 68.68 | 76.05 | 64.28 | - | 20.41 | 63.29 | 64.28 |
| **B+B’** | 31.32 | 23.95 | 35.72 | 100 | 70.59 | 36.71 | 35.72 |

**Figure S2.** Coulombic efficiencies of porous CaFe_2_O_4_ samples: (a) rate performance and (b) subsequent long-term cycling performance of pCFO synthesized at increasing *ϕ* and calcined at 900 ⁰C; (c) rate performance and (d) subsequent long-term cycling performance of pCFO synthesized at *ϕ* = 1.325 and calcined at different temperatures. Because of experimental setup where all cycles begin with a charging phase, expectedly large (> 1000 %) initial efficiencies are omitted in maiden rate performance tests for clarity.

**a)**


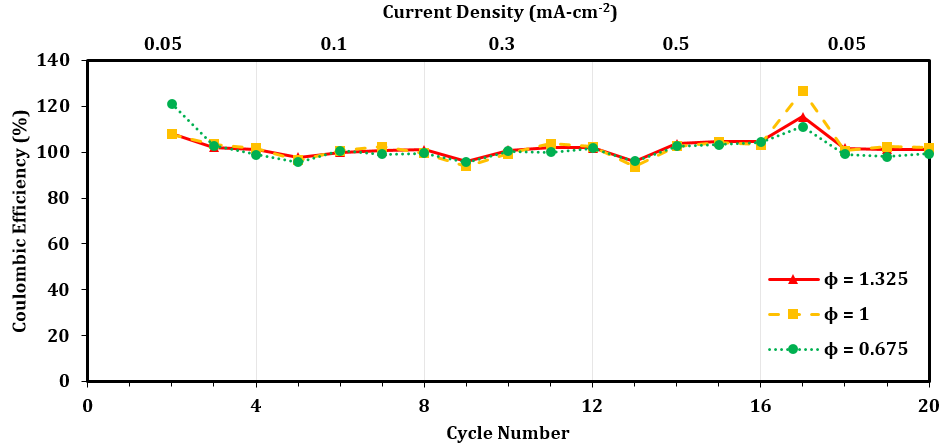


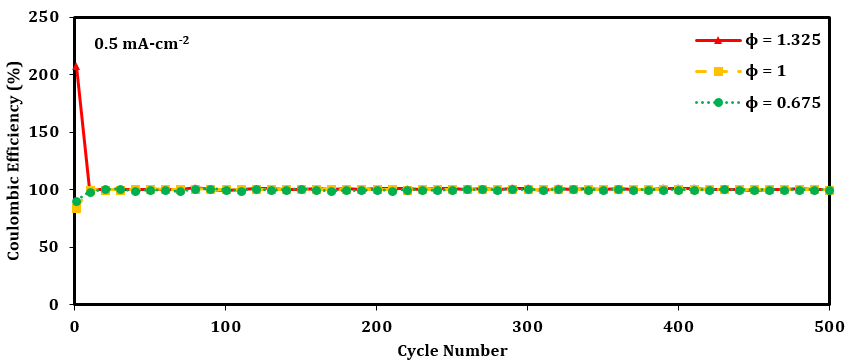


**b)**


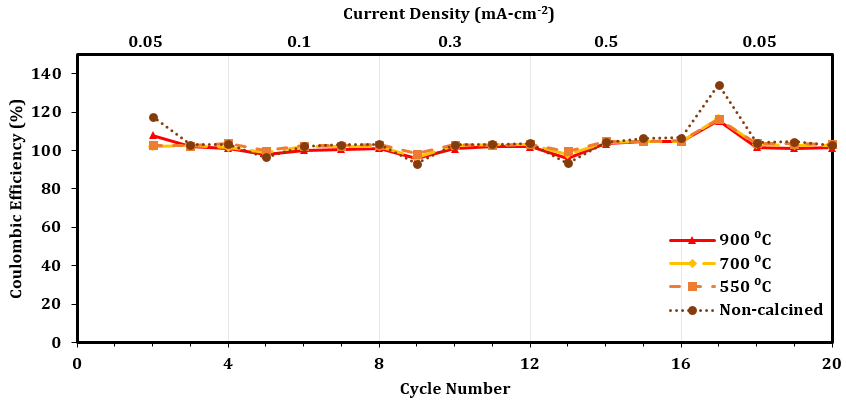


**c)**


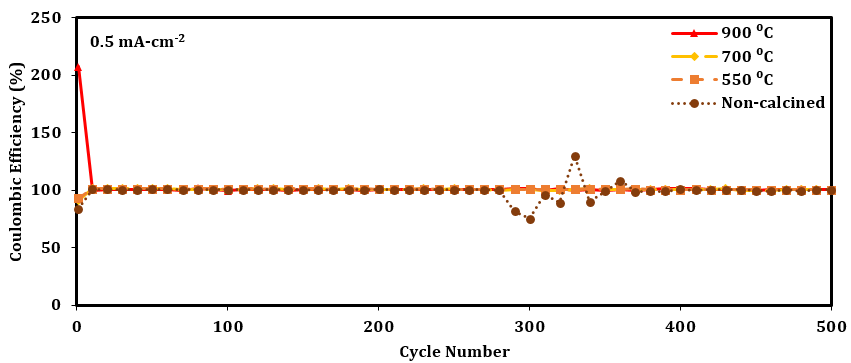


**d)**

**Table S2.** Active mass loadings and current densities for pCFO

|  | **Sample** | **Mass Active Material (mg)** | **Current Density at 0.099 mA**  **(mA g^-1^)** | **Current Density at 0.198 mA**  **(mA g^-1^)** | **Current Density at 0.495 mA**  **(mA g^-1^)** | **Current Density at 0.99 mA**  **(mA g^-1^)** |
| --- | --- | --- | --- | --- | --- | --- |
| **ϕ**  **(calcined 900 ⁰C)** | **0.675** | 1.33 | 74 | 149 | 372 | 744 |
|  | **1** | 1.05 | 94 | 189 | 471 | 943 |
|  | **1.325** | 0.91 | 109 | 218 | 544 | 1088 |
| **Calcination Temperature**  **(ϕ = 1.325)** | **Non-calcined** | 1.05 | 94 | 189 | 471 | 943 |
|  | **550 ⁰C** | 0.77 | 129 | 257 | 643 | 1286 |
|  | **700 ⁰C** | 0.77 | 129 | 257 | 643 | 1286 |
|  | **900 ⁰C** | 0.91 | 109 | 218 | 544 | 1088 |
